# (2,2′-Bipyridine-κ^2^ *N*,*N*′)hydroxido[*N*-(4-tolyl­sulfonyl)­alaninato-κ^2^ *N*,*O* ^1^]copper(II) hemihydrate

**DOI:** 10.1107/S1600536810037529

**Published:** 2010-09-30

**Authors:** Miao-Ling Huang

**Affiliations:** a Department of Chemistry and Science of Life, Quanzhou Normal University, Fujian 362000, People’s Republic of China

## Abstract

In the title complex, [Cu(C_10_H_12_NO_4_S)(OH)(C_10_H_8_N_2_)]·0.5H_2_O, the Cu(II) ion shows a distorted square-pyramidal coordination geometry with two N atoms from the 2,2′-bipyridine ligand and one N and one O atom from the *N*-tosyl-*α*-alaninato ligand forming the basis of the coordination polyhedron and another O atom of the hydroxo group acting as the apex of the pyramid. The solvent water mol­ecule is statistically disordered over two positions.

## Related literature

For related structures of *N*-sulfonyl­ated amino acids as ligands in coordination complexes, see Antolini *et al.* (1985 ▶); Battaglia *et al.* (1983 ▶); Liang *et al.* (2004 ▶); Ma *et al.* (2008 ▶); Menabue & Saladini (1991 ▶). 
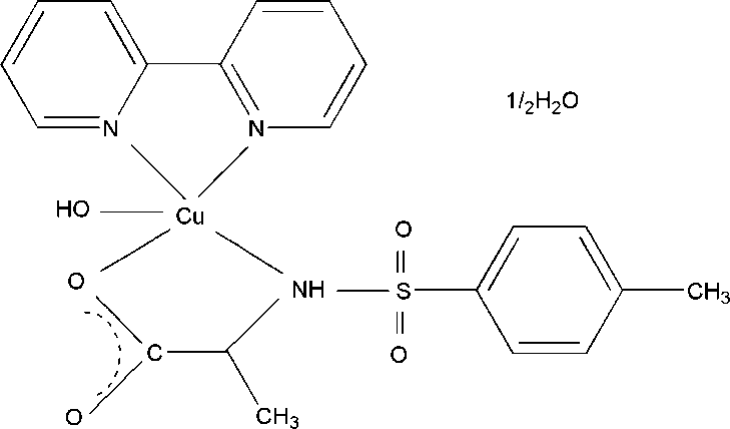


## Experimental

### 

#### Crystal data

[Cu(C_10_H_12_NO_4_S)(OH)(C_10_H_8_N_2_)]·0.5H_2_O
*M* *_r_* = 976.01
Triclinic, 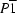

*a* = 7.7246 (13) Å
*b* = 8.3637 (14) Å
*c* = 16.908 (3) Å
α = 103.484 (8)°
β = 95.034 (6)°
γ = 99.656 (5)°
*V* = 1038.0 (3) Å^3^
*Z* = 1
Mo *K*α radiation
μ = 1.19 mm^−1^
*T* = 291 K
0.26 × 0.22 × 0.20 mm

#### Data collection

Bruker SMART APEX CCD diffractometer
Absorption correction: multi-scan (*SADABS*; Sheldrick, 2003 ▶) *T*_min_ = 0.747, *T*_max_ = 0.796
9560 measured reflections
4072 independent reflections
3422 reflections with *I* > 2σ(*I*)
*R*_int_ = 0.035

#### Refinement

*R*[*F* ^2^ > 2σ(*F* ^2^)] = 0.050
*wR*(*F* ^2^) = 0.118
*S* = 1.09
4072 reflections
282 parameters
H-atom parameters constrained
Δρ_max_ = 0.31 e Å^−3^
Δρ_min_ = −0.61 e Å^−3^

### 

Data collection: *SMART* (Bruker, 2001 ▶); cell refinement: *SAINT* (Bruker, 2003 ▶); data reduction: *SAINT*; program(s) used to solve structure: *SHELXS97* (Sheldrick, 2008 ▶); program(s) used to refine structure: *SHELXL97* (Sheldrick, 2008 ▶); molecular graphics: *SHELXTL* (Sheldrick, 2008 ▶); software used to prepare material for publication: *SHELXTL*.

## Supplementary Material

Crystal structure: contains datablocks global, I. DOI: 10.1107/S1600536810037529/im2227sup1.cif

Structure factors: contains datablocks I. DOI: 10.1107/S1600536810037529/im2227Isup2.hkl

Additional supplementary materials:  crystallographic information; 3D view; checkCIF report

## Figures and Tables

**Fig. 1.**
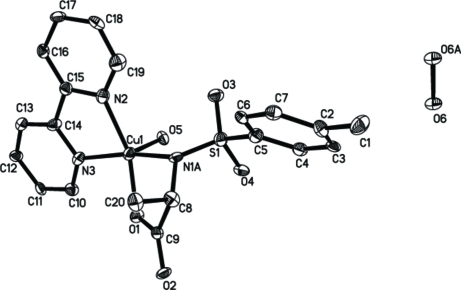
ORTEP drawing of the title compound (I) showing displacement ellipsoids at the 30% probability level. All hydrogen atoms have been omitted for reasons of clarity.

**Fig. 2.**
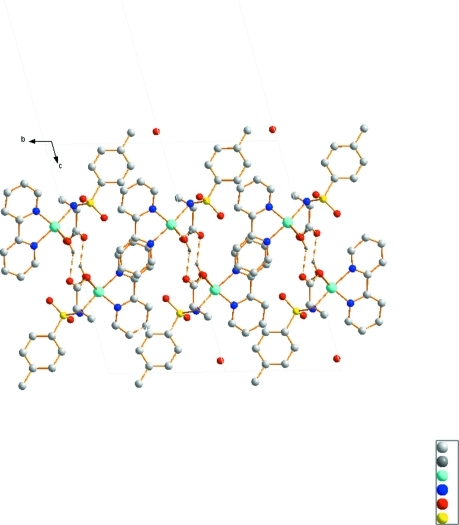
Projection showing the one-dimensional structure formed by H-bonding and π-π stacking interactions of the compound (I).

## supplementary crystallographic information

### Comment

As we know, amino acids play an important role in almost all kinds of biological processes. On the other hand, attachment of an Ar—SO_2_-group at the amino nitrogen of amino acids, such as glycine and β-alanine, increases the number of potential coordination sites of amino acids to three types of O or N donors from carboxyl, sulfoxyl and amino moieties, respectively. This may lead to different coordination modes and has therefore triggered increasing interest in the coordination chemistry of *N*-sulfonyl-amino acids in recent years (Ma, *et al.*, 2008; Liang, *et al.*, 2004; Battaglia, *et al.*, 1983; Menabue & Saladini *et al.*, 1991; Antolini, *et al.*, 1985). In order to continue this research, we synthesized the title complex [Cu(C_10_H_12_NO_4_S)(C_10_H_8_N_2_)(OH)] × 0.5 H_2_O and characterized it by IR and single-crystal X-ray diffraction analyses.

The molecular structure and crystal packing diagram of the title compound are presented in Figs. 1 and 2, respectively. The asymmetric unit of 1 contains one copper cation, one *N*-tosyl-*α*-alaninato ligand, one 2,2'-bipyridine molecule and one coordinated hydroxo group. The coordination geometry around copper may be described as a distorted square pyramid, the basal plane being defined by two N (N2, N3) atoms of 2,2'-bipyridine and one N (N1) and one O (O1) atom of an anionic *N*-tosyl-*α*-alaninato ligand. The apical position is occupied by another O (O5) atom of an hydroxo anion. The Cu—O1 (1.963 (2) Å) and Cu—N1 (2.11 (5) Å) bond distances are longer than those of other N-protected alanine complexes (1.928–1.933 Å) and (1.930–1.956 Å), respectively (Antolini, *et al.*, 1985). Furthermore, the C—O bond distance towards coordinated O1 (1.284 (4) Å) is significantly longer than for non-coordinated O2 (1.235 (4) Å), closely resembling the situation in previously reported complexes (Battaglia, *et al.*, 1983; Antolini, *et al.*, 1985).

It is noteworthy that there are π-π stacking interactions between pyridine rings of 2,2'-bipyridine molecules from adjacent molecules with a centroid distance of 3.314 (4) Å. O–H···O hydrogen bonds between hydroxo groups and carbonyl O atoms of neighboring molecules with donor-acceptor distances of 2.895 (3) and 2.804 (3) Å, respectively are also observed. These two types of intermolecular contacts form a 1-D supramolecular chain in the crystal structure of 1.

### Experimental

To a solution of *DL*-tos-ala (2 mmol) in water-DMF 1:1 (10 ml), an aqueous solution (5 ml) of CuCl_2_ × 2 H_2_O (1 mmol) and a solution of 2,2'-bipyridine (1 mmol) in ethanol (95%, 5 ml) was added. After refluxing for 12 h, the mixture was filtered off while hot. Green single crystals suitable for X-ray analysis were obtained by slow evaporation of the filtrate at room temperature after 35 days (yield 43%). IR (KBr): 3450(*versus*), 1621(*s*), 1601(*s*), 1474(w), 1446(*m*), 1374(w), 1346(w), 1322(*m*), 1258(w), 1161(*s*), 1093(*s*), 983(w), 888(w), 812(w), 775(*s*), 660(*s*), 547(*s*)cm^-1^.

### Refinement

H atoms bonded to C were placed geometrically and treated as riding, (C—H = 0.93–0.96 Å), with *U*_iso_(H) = 1.2 *U*_eq_(C). H atoms bonded to O were found from Fourier difference maps and were refined with restraints for O—H distances (0.8492–0.8612 Å) and *U*_iso_(H) = 1.5 *U*_eq_(O). The H atom bonded to N was also found from Fourier difference maps and were refined without a distance restraint with *U*_iso_(H) = 1.2 *U*_eq_(N).

### Crystal data

**Table:**

[Cu(C_10_H_12_NO_4_S)(OH)(C_10_H_8_N_2_)]·0.5H_2_O	*Z* = 1
*M**_r_* = 976.01	*F*(000) = 504
Triclinic, *P*1	*D*_x_ = 1.561 Mg m^−^^3^
Hall symbol: -P 1	Mo *K*α radiation, λ = 0.71075 Å
*a* = 7.7246 (13) Å	Cell parameters from 1327 reflections
*b* = 8.3637 (14) Å	θ = 2.1–23.2°
*c* = 16.908 (3) Å	µ = 1.19 mm^−^^1^
α = 103.484 (8)°	*T* = 291 K
β = 95.034 (6)°	Block, blue
γ = 99.656 (5)°	0.26 × 0.22 × 0.20 mm
*V* = 1038.0 (3) Å^3^	

### Data collection

**Table:**

Bruker SMART APEX CCD diffractometer	4072 independent reflections
Radiation source: sealed tube	3422 reflections with *I* > 2σ(*I*)
graphite	*R*_int_ = 0.035
phi and ω scans	θ_max_ = 26.0°, θ_min_ = 3.1°
Absorption correction: multi-scan (*SADABS*; Sheldrick, 2003)	*h* = −9→9
*T*_min_ = 0.747, *T*_max_ = 0.796	*k* = −10→10
9560 measured reflections	*l* = −20→20

### Refinement

**Table:**

Refinement on *F*^2^	Primary atom site location: structure-invariant direct methods
Least-squares matrix: full	Secondary atom site location: difference Fourier map
*R*[*F*^2^ > 2σ(*F*^2^)] = 0.050	Hydrogen site location: inferred from neighbouring sites
*wR*(*F*^2^) = 0.118	H-atom parameters constrained
*S* = 1.09	*w* = 1/[σ^2^(*F*_o_^2^) + (0.056*P*)^2^ + 0.9038*P*] where *P* = (*F*_o_^2^ + 2*F*_c_^2^)/3
4072 reflections	(Δ/σ)_max_ < 0.001
282 parameters	Δρ_max_ = 0.31 e Å^−^^3^
0 restraints	Δρ_min_ = −0.61 e Å^−^^3^

### Special details

**Table:**

Geometry. All e.s.d.'s (except the e.s.d. in the dihedral angle between two l.s. planes) are estimated using the full covariance matrix. The cell e.s.d.'s are taken into account individually in the estimation of e.s.d.'s in distances, angles and torsion angles; correlations between e.s.d.'s in cell parameters are only used when they are defined by crystal symmetry. An approximate (isotropic) treatment of cell e.s.d.'s is used for estimating e.s.d.'s involving l.s. planes.
Refinement. Refinement of *F*^2^ against ALL reflections. The weighted *R*-factor *wR* and goodness of fit *S* are based on *F*^2^, conventional *R*-factors *R* are based on *F*, with *F* set to zero for negative *F*^2^. The threshold expression of *F*^2^ > σ(*F*^2^) is used only for calculating *R*-factors(gt) *etc*. and is not relevant to the choice of reflections for refinement. *R*-factors based on *F*^2^ are statistically about twice as large as those based on *F*, and *R*- factors based on ALL data will be even larger.

### Fractional atomic coordinates and isotropic or equivalent isotropic displacement parameters (Å^2^)

**Table:**

	*x*	*y*	*z*	*U*_iso_*/*U*_eq_	Occ. (<1)
C1	0.8537 (6)	0.7675 (5)	1.0458 (3)	0.0446 (10)	
H1A	0.8253	0.8751	1.0463	0.067*	
H1B	0.9785	0.7733	1.0442	0.067*	
H1C	0.8221	0.7357	1.0944	0.067*	
C2	0.7508 (5)	0.6376 (5)	0.9702 (2)	0.0327 (8)	
C3	0.6895 (5)	0.6833 (4)	0.9015 (2)	0.0294 (7)	
H3	0.7098	0.7959	0.9011	0.035*	
C4	0.5956 (5)	0.5611 (4)	0.8307 (2)	0.0302 (7)	
H4	0.5543	0.5932	0.7846	0.036*	
C5	0.5672 (5)	0.3958 (4)	0.8318 (2)	0.0322 (8)	
C6	0.6244 (4)	0.3509 (4)	0.9002 (2)	0.0276 (7)	
H6	0.6030	0.2383	0.9005	0.033*	
C7	0.7152 (4)	0.4706 (5)	0.9703 (2)	0.0301 (7)	
H7	0.7515	0.4373	1.0168	0.036*	
C8	0.7192 (5)	0.1195 (5)	0.7160 (2)	0.0331 (8)	
H8	0.7711	0.2390	0.7363	0.040*	
C9	0.7325 (4)	0.0635 (4)	0.6227 (2)	0.0245 (7)	
C10	0.3590 (5)	−0.3669 (4)	0.4842 (2)	0.0311 (7)	
H10	0.4303	−0.2851	0.4662	0.037*	
C11	0.2866 (5)	−0.5203 (4)	0.4283 (2)	0.0311 (7)	
H11	0.3090	−0.5405	0.3741	0.037*	
C12	0.1819 (5)	−0.6403 (4)	0.4554 (2)	0.0307 (7)	
H12	0.1304	−0.7431	0.4195	0.037*	
C13	0.1538 (5)	−0.6068 (4)	0.5363 (2)	0.0335 (8)	
H13	0.0882	−0.6892	0.5562	0.040*	
C14	0.2237 (5)	−0.4494 (4)	0.5884 (2)	0.0308 (7)	
C15	0.1929 (5)	−0.3953 (4)	0.6758 (2)	0.0291 (7)	
C16	0.0910 (4)	−0.5002 (4)	0.7127 (2)	0.0281 (7)	
H16	0.0389	−0.6098	0.6849	0.034*	
C17	0.0697 (5)	−0.4334 (5)	0.7946 (2)	0.0327 (8)	
H17	0.0056	−0.4995	0.8234	0.039*	
C18	0.1456 (5)	−0.2674 (5)	0.8320 (2)	0.0323 (8)	
H18	0.1302	−0.2208	0.8859	0.039*	
C19	0.2419 (6)	−0.1734 (5)	0.7903 (3)	0.0430 (9)	
H19	0.2916	−0.0625	0.8167	0.052*	
C20	0.8289 (5)	0.0173 (5)	0.7588 (2)	0.0377 (8)	
H20A	0.8086	0.0345	0.8152	0.057*	
H20B	0.9525	0.0536	0.7564	0.057*	
H20C	0.7935	−0.0997	0.7316	0.057*	
Cu1	0.40834 (6)	−0.10913 (5)	0.64296 (3)	0.02818 (14)	
N1	0.5361 (4)	0.0872 (4)	0.73658 (18)	0.0323 (7)	
H1	0.5392	0.0526	0.7886	0.039*	
N2	0.2695 (4)	−0.2328 (4)	0.71207 (18)	0.0302 (6)	
N3	0.3297 (4)	−0.3336 (4)	0.56173 (18)	0.0302 (6)	
O1	0.5991 (3)	−0.0446 (3)	0.58036 (14)	0.0298 (5)	
O2	0.8679 (3)	0.1135 (3)	0.59456 (15)	0.0328 (6)	
O3	0.2660 (3)	0.1975 (3)	0.76155 (16)	0.0355 (6)	
O4	0.4630 (3)	0.3327 (3)	0.67763 (15)	0.0322 (5)	
O5	0.1817 (3)	−0.0315 (3)	0.57743 (15)	0.0319 (5)	
H5A	0.1388	0.0337	0.6106	0.038*	
O6	0.5063 (8)	1.0165 (8)	0.9518 (4)	0.0503 (15)	0.50
H6B	0.5000	1.0000	1.0000	0.060*	
H6D	0.4771	0.9212	0.9177	0.060*	0.50
S1	0.44638 (12)	0.24873 (11)	0.74314 (5)	0.0321 (2)	

### Atomic displacement parameters (Å^2^)

**Table:**

	*U*^11^	*U*^22^	*U*^33^	*U*^12^	*U*^13^	*U*^23^
C1	0.042 (2)	0.040 (2)	0.043 (2)	−0.0102 (18)	−0.0012 (18)	0.0100 (18)
C2	0.0366 (19)	0.0306 (18)	0.0273 (17)	0.0091 (15)	0.0026 (14)	−0.0010 (14)
C3	0.0304 (17)	0.0294 (17)	0.0227 (16)	−0.0004 (14)	0.0140 (13)	−0.0040 (13)
C4	0.0275 (17)	0.0338 (18)	0.0271 (17)	0.0042 (14)	0.0057 (14)	0.0037 (14)
C5	0.0303 (18)	0.0304 (18)	0.0337 (19)	0.0025 (14)	0.0049 (15)	0.0060 (15)
C6	0.0241 (16)	0.0260 (16)	0.0373 (18)	0.0122 (13)	0.0121 (14)	0.0088 (14)
C7	0.0218 (16)	0.0362 (19)	0.0317 (18)	0.0051 (14)	0.0021 (13)	0.0084 (15)
C8	0.0291 (18)	0.0352 (19)	0.0274 (17)	0.0028 (15)	−0.0038 (14)	−0.0019 (15)
C9	0.0251 (16)	0.0242 (16)	0.0264 (16)	0.0077 (13)	0.0047 (13)	0.0082 (13)
C10	0.0328 (18)	0.0312 (18)	0.0251 (17)	0.0039 (14)	0.0048 (14)	0.0002 (14)
C11	0.0390 (19)	0.0258 (16)	0.0319 (18)	0.0165 (15)	0.0114 (15)	0.0044 (14)
C12	0.0270 (17)	0.0282 (17)	0.0327 (18)	−0.0008 (14)	0.0083 (14)	0.0024 (14)
C13	0.039 (2)	0.0256 (17)	0.0378 (19)	0.0042 (15)	0.0107 (16)	0.0104 (15)
C14	0.0249 (16)	0.0294 (17)	0.0312 (18)	−0.0083 (14)	0.0032 (14)	0.0036 (14)
C15	0.0313 (17)	0.0295 (17)	0.0286 (17)	0.0086 (14)	0.0046 (14)	0.0089 (14)
C16	0.0289 (17)	0.0205 (15)	0.0296 (17)	0.0011 (13)	0.0007 (13)	−0.0004 (13)
C17	0.0319 (18)	0.0347 (19)	0.0293 (18)	−0.0020 (15)	0.0142 (14)	0.0062 (15)
C18	0.0286 (18)	0.040 (2)	0.0255 (17)	0.0112 (15)	0.0065 (14)	−0.0026 (15)
C19	0.041 (2)	0.038 (2)	0.041 (2)	−0.0029 (17)	−0.0017 (17)	0.0034 (17)
C20	0.035 (2)	0.0343 (19)	0.039 (2)	0.0070 (16)	−0.0046 (16)	0.0028 (16)
Cu1	0.0297 (2)	0.0226 (2)	0.0300 (2)	0.00039 (16)	0.00412 (16)	0.00544 (16)
N1	0.0294 (15)	0.0388 (17)	0.0254 (14)	0.0103 (13)	0.0039 (12)	−0.0010 (12)
N2	0.0302 (15)	0.0261 (14)	0.0322 (15)	0.0032 (12)	−0.0005 (12)	0.0066 (12)
N3	0.0303 (15)	0.0246 (14)	0.0346 (16)	0.0000 (12)	0.0029 (12)	0.0093 (12)
O1	0.0304 (12)	0.0272 (12)	0.0255 (12)	−0.0030 (10)	0.0013 (10)	0.0014 (10)
O2	0.0292 (13)	0.0290 (12)	0.0360 (13)	−0.0079 (10)	0.0113 (10)	0.0070 (10)
O3	0.0299 (13)	0.0384 (14)	0.0357 (14)	−0.0002 (11)	0.0080 (11)	0.0081 (11)
O4	0.0275 (12)	0.0432 (14)	0.0314 (13)	0.0119 (11)	0.0123 (10)	0.0137 (11)
O5	0.0362 (13)	0.0283 (12)	0.0333 (13)	0.0144 (10)	0.0066 (11)	0.0053 (10)
O6	0.045 (3)	0.060 (4)	0.054 (4)	0.004 (3)	0.020 (3)	0.029 (3)
S1	0.0347 (5)	0.0279 (4)	0.0305 (4)	0.0019 (4)	0.0049 (4)	0.0038 (4)

### Geometric parameters (Å, °)

**Table:**

C1—C2	1.526 (5)	C13—H13	0.9300
C1—H1A	0.9600	C14—N3	1.347 (4)
C1—H1B	0.9600	C14—C15	1.493 (5)
C1—H1C	0.9600	C15—N2	1.361 (4)
C2—C3	1.376 (5)	C15—C16	1.375 (5)
C2—C7	1.378 (5)	C16—C17	1.401 (5)
C3—C4	1.425 (5)	C16—H16	0.9300
C3—H3	0.9300	C17—C18	1.388 (5)
C4—C5	1.367 (5)	C17—H17	0.9300
C4—H4	0.9300	C18—C19	1.351 (6)
C5—C6	1.357 (5)	C18—H18	0.9300
C5—S1	1.772 (4)	C19—N2	1.349 (5)
C6—C7	1.402 (5)	C19—H19	0.9300
C6—H6	0.9300	C20—H20A	0.9600
C7—H7	0.9300	C20—H20B	0.9600
C8—N1	1.482 (5)	C20—H20C	0.9600
C8—C20	1.549 (5)	Cu1—O1	1.964 (2)
C8—C9	1.555 (5)	Cu1—N2	1.999 (3)
C8—H8	0.9792	Cu1—N3	2.012 (3)
C9—O2	1.236 (4)	Cu1—N1	2.036 (3)
C9—O1	1.284 (4)	Cu1—O5	2.257 (2)
C10—N3	1.323 (5)	N1—S1	1.605 (3)
C10—C11	1.398 (5)	N1—H1	0.9871
C10—H10	0.9300	O3—S1	1.470 (3)
C11—C12	1.371 (5)	O4—S1	1.447 (3)
C11—H11	0.9300	O5—H5A	0.8195
C12—C13	1.375 (5)	O6—O6^i^	1.722 (11)
C12—H12	0.9300	O6—H6B	0.8612
C13—C14	1.392 (5)	O6—H6D	0.8492
			
C2—C1—H1A	109.5	C15—C16—C17	116.5 (3)
C2—C1—H1B	109.5	C15—C16—H16	121.7
H1A—C1—H1B	109.5	C17—C16—H16	121.7
C2—C1—H1C	109.5	C18—C17—C16	119.1 (3)
H1A—C1—H1C	109.5	C18—C17—H17	120.4
H1B—C1—H1C	109.5	C16—C17—H17	120.4
C3—C2—C7	118.5 (3)	C19—C18—C17	120.0 (3)
C3—C2—C1	121.5 (3)	C19—C18—H18	120.0
C7—C2—C1	120.0 (3)	C17—C18—H18	120.0
C2—C3—C4	121.1 (3)	N2—C19—C18	123.1 (4)
C2—C3—H3	119.4	N2—C19—H19	118.4
C4—C3—H3	119.4	C18—C19—H19	118.4
C5—C4—C3	119.0 (3)	C8—C20—H20A	109.5
C5—C4—H4	120.5	C8—C20—H20B	109.5
C3—C4—H4	120.5	H20A—C20—H20B	109.5
C6—C5—C4	120.0 (3)	C8—C20—H20C	109.5
C6—C5—S1	122.6 (3)	H20A—C20—H20C	109.5
C4—C5—S1	117.3 (3)	H20B—C20—H20C	109.5
C5—C6—C7	121.4 (3)	O1—Cu1—N2	159.44 (11)
C5—C6—H6	119.3	O1—Cu1—N3	91.52 (11)
C7—C6—H6	119.3	N2—Cu1—N3	80.58 (12)
C2—C7—C6	120.0 (3)	O1—Cu1—N1	86.23 (11)
C2—C7—H7	120.0	N2—Cu1—N1	96.46 (12)
C6—C7—H7	120.0	N3—Cu1—N1	164.93 (12)
N1—C8—C20	107.7 (3)	O1—Cu1—O5	100.11 (10)
N1—C8—C9	113.9 (3)	N2—Cu1—O5	98.22 (11)
C20—C8—C9	106.9 (3)	N3—Cu1—O5	85.79 (11)
N1—C8—H8	109.8	N1—Cu1—O5	109.28 (11)
C20—C8—H8	109.3	C8—N1—S1	111.6 (3)
C9—C8—H8	109.0	C8—N1—Cu1	103.8 (2)
O2—C9—O1	124.2 (3)	S1—N1—Cu1	111.98 (16)
O2—C9—C8	120.9 (3)	C8—N1—H1	108.8
O1—C9—C8	114.7 (3)	S1—N1—H1	110.9
N3—C10—C11	122.5 (3)	Cu1—N1—H1	109.5
N3—C10—H10	118.8	C19—N2—C15	116.3 (3)
C11—C10—H10	118.8	C19—N2—Cu1	127.5 (3)
C12—C11—C10	118.4 (3)	C15—N2—Cu1	116.2 (2)
C12—C11—H11	120.8	C10—N3—C14	119.5 (3)
C10—C11—H11	120.8	C10—N3—Cu1	124.9 (2)
C11—C12—C13	119.2 (3)	C14—N3—Cu1	115.2 (2)
C11—C12—H12	120.4	C9—O1—Cu1	115.1 (2)
C13—C12—H12	120.4	Cu1—O5—H5A	109.6
C12—C13—C14	119.9 (3)	O6^i^—O6—H6D	107.0
C12—C13—H13	120.1	H6B—O6—H6D	107.0
C14—C13—H13	120.1	O4—S1—O3	116.61 (15)
N3—C14—C13	120.4 (3)	O4—S1—N1	116.92 (16)
N3—C14—C15	114.9 (3)	O3—S1—N1	105.54 (16)
C13—C14—C15	124.6 (3)	O4—S1—C5	105.06 (16)
N2—C15—C16	124.9 (3)	O3—S1—C5	107.62 (16)
N2—C15—C14	113.0 (3)	N1—S1—C5	104.07 (17)
C16—C15—C14	122.1 (3)		
			
C7—C2—C3—C4	−1.7 (5)	C14—C15—N2—C19	177.8 (3)
C1—C2—C3—C4	179.1 (3)	C16—C15—N2—Cu1	−179.3 (3)
C2—C3—C4—C5	−0.3 (5)	C14—C15—N2—Cu1	−1.8 (4)
C3—C4—C5—C6	1.6 (5)	O1—Cu1—N2—C19	111.7 (4)
C3—C4—C5—S1	178.9 (2)	N3—Cu1—N2—C19	−179.7 (3)
C4—C5—C6—C7	−0.9 (5)	N1—Cu1—N2—C19	15.2 (3)
S1—C5—C6—C7	−178.1 (3)	O5—Cu1—N2—C19	−95.4 (3)
C3—C2—C7—C6	2.4 (5)	O1—Cu1—N2—C15	−68.8 (4)
C1—C2—C7—C6	−178.5 (3)	N3—Cu1—N2—C15	−0.3 (2)
C5—C6—C7—C2	−1.1 (5)	N1—Cu1—N2—C15	−165.3 (2)
N1—C8—C9—O2	164.5 (3)	O5—Cu1—N2—C15	84.1 (2)
C20—C8—C9—O2	−76.6 (4)	C11—C10—N3—C14	−1.3 (5)
N1—C8—C9—O1	−19.9 (4)	C11—C10—N3—Cu1	−173.6 (3)
C20—C8—C9—O1	98.9 (3)	C13—C14—N3—C10	3.8 (5)
N3—C10—C11—C12	−0.1 (5)	C15—C14—N3—C10	−177.1 (3)
C10—C11—C12—C13	−0.9 (5)	C13—C14—N3—Cu1	176.8 (3)
C11—C12—C13—C14	3.4 (6)	C15—C14—N3—Cu1	−4.1 (4)
C12—C13—C14—N3	−4.9 (6)	O1—Cu1—N3—C10	−24.1 (3)
C12—C13—C14—C15	176.1 (4)	N2—Cu1—N3—C10	175.0 (3)
N3—C14—C15—N2	3.8 (5)	N1—Cu1—N3—C10	−105.2 (5)
C13—C14—C15—N2	−177.1 (3)	O5—Cu1—N3—C10	76.0 (3)
N3—C14—C15—C16	−178.6 (3)	O1—Cu1—N3—C14	163.4 (3)
C13—C14—C15—C16	0.4 (6)	N2—Cu1—N3—C14	2.5 (3)
N2—C15—C16—C17	−1.6 (5)	N1—Cu1—N3—C14	82.2 (5)
C14—C15—C16—C17	−178.9 (3)	O5—Cu1—N3—C14	−96.6 (3)
C15—C16—C17—C18	2.2 (5)	O2—C9—O1—Cu1	176.8 (3)
C16—C17—C18—C19	−1.5 (6)	C8—C9—O1—Cu1	1.3 (4)
C17—C18—C19—N2	0.1 (6)	N2—Cu1—O1—C9	−86.9 (4)
C20—C8—N1—S1	147.0 (2)	N3—Cu1—O1—C9	−153.6 (2)
C9—C8—N1—S1	−94.7 (3)	N1—Cu1—O1—C9	11.5 (2)
C20—C8—N1—Cu1	−92.3 (3)	O5—Cu1—O1—C9	120.4 (2)
C9—C8—N1—Cu1	26.1 (3)	C8—N1—S1—O4	47.1 (3)
O1—Cu1—N1—C8	−20.3 (2)	Cu1—N1—S1—O4	−68.8 (2)
N2—Cu1—N1—C8	139.2 (2)	C8—N1—S1—O3	178.6 (2)
N3—Cu1—N1—C8	61.5 (5)	Cu1—N1—S1—O3	62.71 (19)
O5—Cu1—N1—C8	−119.7 (2)	C8—N1—S1—C5	−68.2 (3)
O1—Cu1—N1—S1	100.20 (17)	Cu1—N1—S1—C5	175.89 (16)
N2—Cu1—N1—S1	−100.27 (18)	C6—C5—S1—O4	−161.3 (3)
N3—Cu1—N1—S1	−178.0 (4)	C4—C5—S1—O4	21.4 (3)
O5—Cu1—N1—S1	0.8 (2)	C6—C5—S1—O3	73.8 (3)
C18—C19—N2—C15	0.6 (6)	C4—C5—S1—O3	−103.5 (3)
C18—C19—N2—Cu1	−180.0 (3)	C6—C5—S1—N1	−37.9 (3)
C16—C15—N2—C19	0.3 (5)	C4—C5—S1—N1	144.8 (3)

Symmetry codes: (i) −*x*+1, −*y*+2, −*z*+2.
